# Shared Medical Appointments and Mindfulness for Type 2 Diabetes—A Mixed-Methods Feasibility Study

**DOI:** 10.3389/fendo.2020.570777

**Published:** 2020-10-06

**Authors:** Carolyn Ee, Barbora de Courten, Nicole Avard, Michael de Manincor, Mahmoud A. Al-Dabbas, Jie Hao, Kate McBride, Shamieka Dubois, Rhiannon Lee White, Catharine Fleming, Garry Egger, Angela Blair, John Stevens, Freya MacMillan, Gary Deed, Suzanne Grant, Kate Templeman, Dennis Chang

**Affiliations:** ^1^ NICM Health Research Institute, Western Sydney University, Penrith, NSW, Australia; ^2^ Translational Health Research Institute, Western Sydney University, Penrith, NSW, Australia; ^3^ Department of Medicine, School of Clinical Sciences, Monash University, Melbourne, VIC, Australia; ^4^ Next Practice Health, Erina, Sydney, NSW, Australia; ^5^ School of Medicine, Western Sydney University, Penrith, NSW, Australia; ^6^ School of Health Sciences, Western Sydney University, Penrith, NSW, Australia; ^7^ School of Health and Human Sciences, Southern Cross University, Lismore, NSW, Australia; ^8^ Diabetes NSW and ACT, Sydney, NSW, Australia; ^9^ Mediwell Clinic, Brisbane, QLD, Australia

**Keywords:** type 2 diabetes, primary care, shared medical appointments, mindfulness, glycemic control, feasibility study, pSMAs for type 2 diabetes

## Abstract

**Introduction:**

Type 2 diabetes (T2DM) is a major health concern with significant personal and healthcare system costs. There is growing interest in using shared medical appointments (SMAs) for management of T2DM. We hypothesize that adding mindfulness to SMAs may be beneficial. This study aimed to assess the feasibility and acceptability of SMAs with mindfulness for T2DM within primary care in Australia.

**Materials and Methods:**

We conducted a single-blind randomized controlled feasibility study of SMAs within primary care for people with T2DM living in Western Sydney, Australia. People with T2DM, age 21 years and over, with HbA1c > 6.5% or fasting glucose >7.00 mmol/L within the past 3 months were eligible to enroll. The intervention group attended six 2-h programmed SMAs (pSMAs) which were held fortnightly. pSMAs included a structured education program and mindfulness component. The control group received usual care from their healthcare providers. We collected quantitative and qualitative data on acceptability as well as glycemic control (glycated hemoglobin and continuous glucose monitoring), lipids, anthropometric measures, blood pressure, self-reported psychological outcomes, quality of life, diet, and physical activity using an ActiGraph accelerometer.

**Results:**

Over a 2-month period, we enrolled 18 participants (10 females, 8 males) with a mean age of 58 years (standard deviation 9.8). We had 94.4% retention. All participants in the intervention group completed at least four pSMAs. Participants reported that attending pSMAs had been a positive experience that allowed them to accept their diagnosis and empowered them to make changes, which led to beneficial effects including weight loss and better glycemic control. Four pSMA participants found the mindfulness component helpful while two did not. All of the seven participants who contributed to qualitative evaluation reported improved psychosocial wellbeing and found the group setting beneficial. There was a significant difference in total cholesterol levels at 12 weeks between groups (3.86 mmol/L in intervention group vs. 4.15 mmol/L in the control group; *p* = 0.025) as well as pain intensity levels as measured by the PROMIS-29 (2.11 vs. 2.38; *p* = 0.034).

**Conclusion:**

pSMAs are feasible and acceptable to people with T2DM and may result in clinical improvement. A follow-up fully-powered randomized controlled trial is warranted.

**Clinical Trial Registration:**

Australia and New Zealand Clinical Trial Registry, identifier ACTRN12619000892112.

## Introduction

Diabetes is a major cause of morbidity and mortality globally. An estimated 8.5% of the world’s population lives with diabetes, and diabetes was the direct cause of 1.6 million deaths, or 11% of all deaths, in 2014 worldwide (1). In 2019, global health expenditure on diabetes was estimated at USD$760 billion (2). In Australia, type 2 diabetes (T2DM) is diagnosed once every 5 min (3) and the number of diagnoses is expected to double by 2033 to 3.5 million (4). T2DM is a National Health Priority Area (5) with an annual financial cost of AUD $14.6 billion (3). Health consequences of T2DM are devastating and include stroke and coronary heart disease, blindness, renal failure, neuropathy, and peripheral vascular disease (4). Moreover, T2DM is one of the leading causes of morbidity and mortality in Australia and commonly causes psychological distress (4). It is estimated that every 1% reduction in HbA1c significantly reduces risk of T2DM related deaths (−21%) and microvascular complications (−37%) (6).

There is growing interest in the possible role of shared medical appointments (SMAs) in managing chronic disease (7). SMAs are defined as “*consecutive individual medical visits carried out in a supportive group setting of similar patients where all can listen, interact, and learn*” and may have the advantages of being cost-effective, encouraging peer support, improving clinician satisfaction, and reducing repetition of health information (8). SMAs involve a medical practitioner [e.g. general practitioner (GP) or specialist] consulting with patients sequentially among a group of patients who can interact throughout the consultation under the guidance and direction of a trained facilitator (usually a practice nurse or other allied health professional). A variant of SMAs is the programmed SMA or pSMA, which incorporates a structured educational component. SMAs are also a time-efficient way of providing chronic disease management, and are becoming increasingly popular within primary care (9).

The evidence on SMAs for T2DM is inconsistent, mostly due to heterogeneity of the interventions and sample characteristics; however, there is some evidence for reduction of HbA1c, weight, blood pressure, lipids, and improved quality of life and patient satisfaction (10). SMAs are also cost-effective compared to one-on-one care (8). There is a compelling need to further explore the role of SMAs in the management of diabetes and its complications as well as resulting psychological distress within primary care. Of note, there are no published randomized controlled trials evaluating the effectiveness of SMAs for T2DM in Australia.

Mindfulness-based interventions may be beneficial for quality of life, anxiety, and depression in people with T2DM but the evidence for physiological outcome changes is inconsistent (11). However, mindfulness meditation may play an important adjunctive role in managing other metabolic conditions. For example, there is evidence from systematic reviews that mindfulness meditation in the general population can improve eating behaviors and increase physical activity (11–16) by teaching participants to become more accepting of the physical discomfort of portion control and physical exercise and is effective for weight loss.

SMAs may therefore represent an innovative model of care that could improve glycemic control in people with T2DM and enhance T2DM management within primary care; however, more robust evidence is required. Prior to undertaking expensive randomized controlled trials, feasibility studies are recommended in order to assess the likelihood of successful recruitment of a fully-powered trial as well as the acceptability of trial procedures to participants. To this end, we conducted a mixed-methods study evaluating the feasibility and acceptability of pSMAs for people with T2DM. We hypothesized that including a mindfulness component to pSMAs would improve adherence to lifestyle recommendations and facilitate healthier behaviors.

## Materials and Methods

The primary objective for the study was to assess recruitment, retention, and adherence rates as well as acceptability of trial procedures. Secondary objectives were to determine an effect size for pSMAs vs. usual care and examine for a trend between groups for change in glycemic control, blood pressure, lipids, anthropometric measures, quality of life, psychological outcomes, and lifestyle habits.

### Study Design

This was a mixed-methods study that included a prospective parallel pragmatic single-blind randomized controlled feasibility trial. Feasibility was also assessed using qualitative methods (a focus group and semi-structured interviews).

Ethics approval was granted by the Western Sydney University Human Research Ethics Committee (H12925 16/11/2018). The trial was registered on the Australian New Zealand Clinical Trials Registry (ACTRN12619000892112).

### Randomization and Blinding

Participants were randomized in a 1:1 ratio to receive either the intervention (pSMA) or usual care. The randomization sequence was created using a computer program (www.sealedenvelope.com) by a researcher external to the research team. Permuted blocks of six were used. Allocation was concealed using consecutively number sealed opaque envelope. Before opening each envelope, the research assistant wrote the participant’s name on the envelope, the date, and signed the envelope as a record of randomization. Investigators (apart from the research assistant who implemented the randomization process) were blinded to treatment allocation but participants were not. The research assistant who collected anthropometric measurement collection (JH) was also blinded to allocation.

### Sample Size

As this was a feasibility study, a sample size calculation was not required. SMAs typically involve 10–12 participants per group. We aimed to randomize 24 participants altogether (12 to a pSMA group, 12 to usual care).

### Participants

The study took place in an academic primary care clinic setting (Western Sydney Integrative Health—an academic clinic within Western Sydney University) in Sydney.

Recruitment began on 12^th^ March 2019 and ended on 15^th^ May 2019. The study was advertised on Diabetes NSW & ACT social media pages on 15^th^ March and in the Diabetes NSW & ACT newsletter in 4 April. The study was advertised on Facebook from 19^th^ March for six days and on Google from 23^rd^ March for 7 days. Other promotion channels were the Western Sydney University online staff newsletter on 12^th^ March and 16^th^ April 2019, and the India Club newsletter 4^th^ April 2019 (a voluntary organization established to promote the interests of the Indian Australian community).

Participants were eligible if they were aged over 21 years, lived in the Greater Western Sydney region in Australia, had a diagnosis of T2DM, and had evidence of HbA1c > 6.5% or fasting blood glucose > 7.00 mmol/L within the preceding 3 months. Exclusion criteria were:

- Unable to attend for pSMAs;
- Pregnant or planning pregnancy in the next 3 months;
- Serious medical or psychological conditions (e.g., metastatic cancer and poorly controlled schizophrenia);
- Not fluent in English (unable to follow conversations and instructions in English and unable to read English); and
- Known allergy to medical adhesives (because of the risk of allergy to adhesive from the continuous glucose monitoring system)

### Outcomes

Primary outcome measures were recruitment rate, retention rate, and adherence rate. Acceptability was also assessed by an exit questionnaire and qualitative methods.

We used the following to define our feasibility measures:

- Recruitment rates: number of enquiries and number of enrolments per month of active recruitment; percentage conversion to enrolment measured as *n* enrolled/*n* of enquiries, and *n* enrolled/*n* potentially eligible.
- Retention rate: *n* completing 12-week intervention and outcome measures/*n* enrolled.
- Adherence rate: *n* completing at least 4 of 6 PSMAs/total *n* allocated to the intervention group.

Secondary outcome measures were:

- Glycemic management (from HbA1c, and also time in range/mean glucose measured by a 14-day FreeStyle Libre Continuous Glucose Monitor);
- Fasting lipids;
- Anthropometric measures (weight, body mass index/BMI, waist and hip circumference, and waist/hip circumference ratio);
- Blood pressure;
- Anxiety and depression using the Beck Depression Inventory (BDI) (17) and State Anxiety Index (SAI) (18);
- Diabetes-related distress using the Problem Areas in Diabetes (PAID) scale (19);
- Generic quality of life and wellbeing using the EuroQol-5D (EQ5D) (20) and Patient-Reported Outcomes Information System questionnaire (PROMIS-29) (21);
- Self-reported diet quality using three questions about fruit, vegetable and takeaway intake taken from the NSW Population Health Survey Questionnaire 2017, which is an annual telephone-based survey of 15,000 adults in the state of New South Wales, Australia (22);
- Physical activity levels as measured by an ActiGraph accelerometer (minutes per week of light, moderate and vigorous activity; number of steps per day; number of sedentary hours per day); and
- Number of minutes of mindfulness practice per week (measured using a home meditation log)

#### Pre-Intervention and Baseline Visit Data Collection

At a pre-intervention visit, a research assistant obtained written informed consent and details on concomitant medications and past medical history. Weight was measured to the nearest 0.1 kg using a calibrated medical-grade digital scale (Seca 803) and waist and hip circumference to the nearest 0.1cm using a medical-grade steel tape measure (Seca 201). Height was measured using a wall-mounted stadiometer. Anthropometric measures and blood pressure were measured according to standard procedures (23). Participants completed a demographic and medical questionnaire that collected information about age, ethnicity, education, cigarette use, year of diagnosis of T2DM, and presence of complications of diabetes. Study data were collected and managed using REDCap electronic data capture tools hosted at Western Sydney University (24, 25). REDCap (Research Electronic Data Capture) is a secure, web-based software platform designed to support data capture for research studies, providing: 1) an intuitive interface for validated data capture; 2) audit trails for tracking data manipulation and export procedures; 3) automated export procedures for seamless data downloads to common statistical packages; and 4) procedures for data integration and interoperability with external sources.

A 14-day FreeStyle Libre Continuous Glucose Monitor sensor was inserted into the posterior upper arm. We asked participants to wear an ActiGraph accelerometer (GT3X, LLC, Fort Walton Beach, FL) on their right hip for 5–7 days to measure light, moderate, and vigorous physical activity, as well as their number of steps per day and time spent in sedentary behavior. A sufficient number of days are needed for the resulting average MVPA per day to reflect the participants usual PA level. However, it is equally important to not overburden participants. Evidence across multiple accelerometer studies shows that at least 3 days of PA data are needed to reliably estimate usual or habitual PA levels (26). As accelerometer compliance is sometimes low, asking participants to wear the device for 5–7 consecutive days is likely to ensure at least three valid days of PA data, without overburdening participants more than necessary. We removed non-wear-time from the participants’ data files by identifying strings of consecutive zero count-values lasting ≥60 min, while allowing for a 1- to 2-min spike tolerance of counts between 0 and 100. After removing wear-time, a calendar day was considered valid if the accelerometer was worn for ≥10 h, and participants with at least 3 valid days were included in the analysis. Using established cut-points we calculated participants’ sedentary, light, moderate, and vigorous physical activity (27).

Participants attended for blood collection at private pathology centres (Laverty Pathology) after an overnight fast, a few days prior to the baseline visit. Whole blood was analyzed for glycated haemoglobin using ion-exchange high-performance liquid chromatography/HPLC (D-100™ HbA_1c_ test; Bio-Rad Laboratories). Serum was analyzed for total cholesterol using the enzymatic method (ADVIA^®^ Chemistry Concentrated Cholesterol Reagent/CHOL_c; ADVIA^®^ Chemistry Systems) and for triglycerides using the Fossati three-step enzymatic reaction with a Trinder endpoint (ADVIA^®^ Chemistry Triglycerides_2 Concentrated/TIRG_c assay; ADVIA^®^ Chemistry Systems).

At the baseline visit, the Freestyle Libre sensor was removed and disposed of, and data was downloaded from the sensor. The accelerometer was collected from the participant, and any adverse events that had been experienced were recorded.

#### End of Treatment Data Collection

Participants attended a Week 12 clinic visit during which adverse events that were reported were recorded and concomitant medications and medical history were updated. Anthropometric measures, blood pressure, and PROMs were collected as per the pre-intervention visit. Participants also completed an exit survey about acceptability of trial procedures; however, due to an administrative error only the first question was completed at Week 12, with the remainder of the questions completed in December 2019 when the research team discovered the missing data. Participants were fitted with another 14-day FreeStyle Libre Continuous Glucose Monitor sensor and wore that for 14 days, then returned for removal of the sensor. Participants were provided with an accelerometer to wear for 5–7 days and had further testing for HbA1c and fasting lipids.246.

### Intervention: Programmed Shared Medical Appointments With Mindfulness

The intervention was a series of face-to-face pSMAs with an adjunctive mindfulness meditation component. The frequency, length and duration of pSMAs is based on a study by Egger et al. on weight loss for people with obesity (9) as well as from reviewing the literature. The frequency of SMAs for people with T2DM in other studies has varied from every 3 weeks to every 3 months, with more frequent SMAs being generally associated with improved outcomes for weight loss. The feedback from study by Egger et al. was that monthly SMAs were too infrequent for weight loss, and fortnightly SMAs were feasible. Most SMAs are between 6-12 people of an optimum of six sessions based on the earlier GutBuster’s Program by Egger (28).

The 12-week program included six fortnightly 2-h pSMAs delivered by a GP, facilitator (accredited diabetes educator), documenter, and a meditation teacher. The pSMA ran as follows:

- A confidentiality agreement was signed by all participants at the beginning of the first SMA;
- Each participant was asked for their questions for the GP, and these were written down on a whiteboard;
- 60 min of SMAs where the GP consulted with each participant consecutively for approximately 5–7 min at a time per individual consultation;
- 30 min of structured education delivered by the facilitator/diabetes educator. These were made interactive through the use of props and handouts, for example, food models, nail files, and fact sheets from the National Diabetes Services Scheme, an initiative of Diabetes Australia (Australia’s national body for people affected by diabetes); and
- 20 min of guided mindfulness meditation delivered by a meditation teacher (see **Appendix 2** for more information).

For quality assurance, and to ensure fidelity of the intervention, a researcher (CE) observed one of the pSMAs and provided corrective feedback as appropriate.

Eight topics on diabetes education were offered to the participants who were then able to choose the six topics that they wished to have delivered (see **Appendix 1** for details on the education program). Participants were encouraged to set their own lifestyle goals based on the RACGP handbook on general practice management of T2DM (29). Key goals included weight loss of 5%.

The mindfulness component was delivered by an experienced meditation teacher and consisted of 15–20 min of guided mindfulness meditation. Mindfulness was defined as “paying attention in a particular way; on purpose, in the present moment, and nonjudgementally” (30). Each guided mindfulness session was recorded and made available for home practice. Participants were encouraged to practice mindfulness meditation at home and to record it in meditation logs. More details are available in **Appendix 1**.

### Control

Participants allocated to the control group received usual care only, defined as care as provided by their usual GP. They were encouraged to register for the GetHealthy NSW program (31), which is a free telephone-based lifestyle coaching service, and to use the free Smiling Mind (32) mindfulness meditation app regularly.

### Prior and Concomitant Medications

There were no restrictions on concomitant medications in this trial. Concomitant medications were monitored using a Concomitant Medication Log.

### Participant Reimbursement

All participants were reimbursed AUD$50 in total after attending the 12-week visit, for travel costs associated with the study.

### Qualitative Interviews

We conducted a qualitative evaluation of the acceptability and feasibility of the pSMA program. Participants who received the pSMAs intervention were invited to provide feedback on the acceptability of the intervention in a 90 to 120-min focus group or a one-on-one, semi-structured interview.

The focus group and interviews were conducted by researchers external to the main study (SD and KM) so as to not influence participant responses in any way. Key questions in the interview schedule included feedback on the content, structure and frequency of the pSMAs, perceived value of the mindfulness component, and suggestions for improvement. Focus groups and interviews were audio-recorded and transcribed verbatim by a professional transcribing service.

### Statistical Methods and Data Analysis

Recruitment, retention, and adherence rates are presented with descriptive statistics. We used ANOVA to identify between-group differences, adjusting for baseline score as a co-variate. Where data was not normally distributed, we used non-parametric measures (Mann-Whitney U tests). Intention-to-treat analysis was used. Analysis and interpretation of the findings was conducted by blinded investigators. Investigators agreed on the interpretation of the findings before revealing the allocation of either group. At the time of interpretation of findings, the two groups were only referred to as “Group 1” or “Group 2”. Group numbers were also not revealed as this could have compromised blinding.

The de-identified transcripts were analyzed using thematic analysis. Thematic analysis encompasses identifying and analyzing reoccurring patterns of significance in regard to the research question being addressed (33). Themes and sub-themes meaningful to the research matter emerged as a result of these reoccurring patterns within the data. Transcripts were coded using Quirkos v.1.5.1 software (34). Participant excerpts are included in the results labelled by a participant identification number to highlight key themes and sub-themes.

## Results

### Recruitment

Facebook advertisement received 11,576 total impressions with an engagement rate of 12% and CPR of $0.69c, at a total cost of AUD$100 over six days. The Google advertisements generated 144 clicks and 4,320 impressions at a cost of AUD$380 over 7 days.

**Figure 1:**
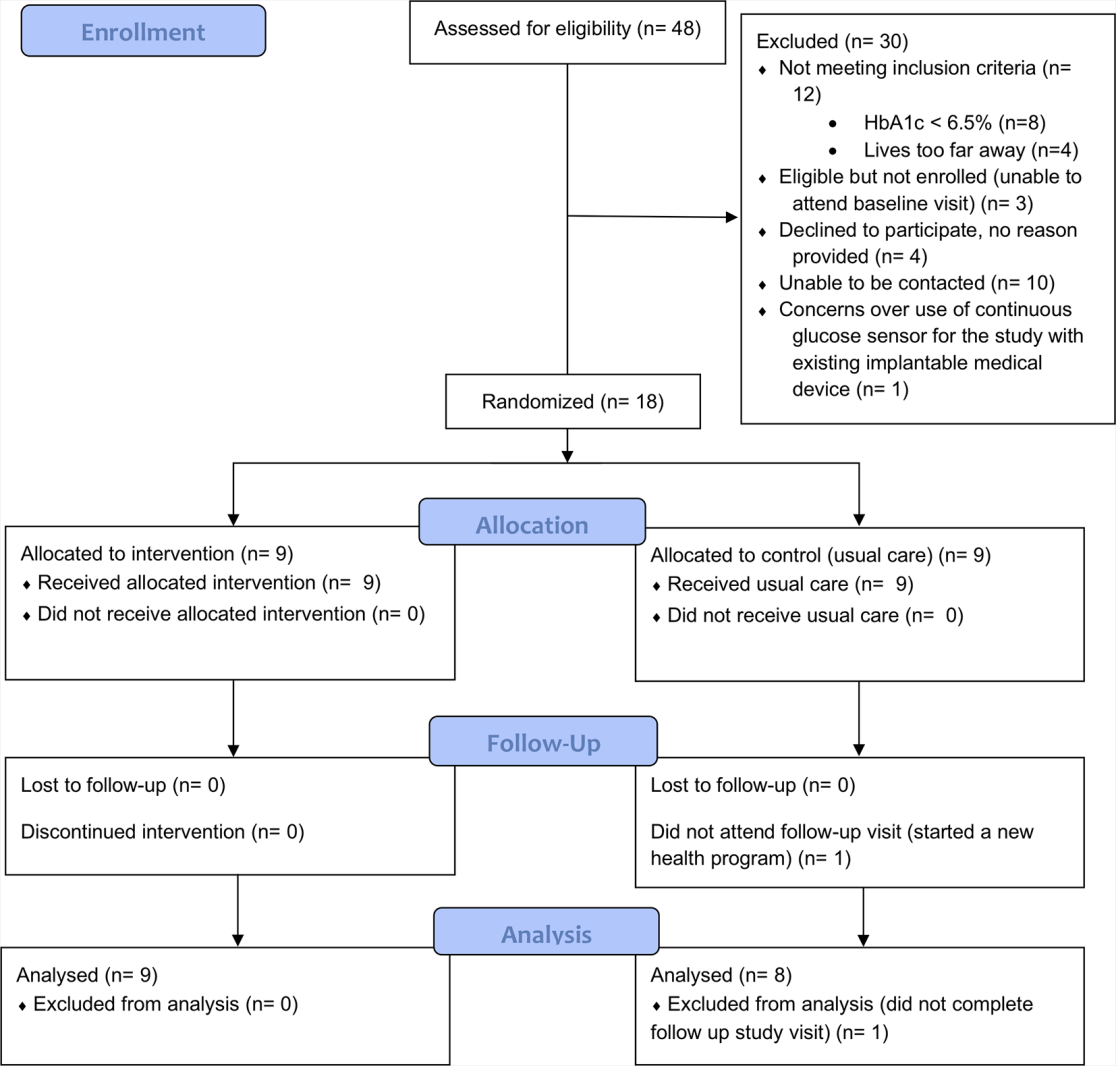
CONSORT flowchart of participant flow.

**Figure 1** describes participant flow through the trial.

### Baseline Data

**Tables 1**, **2** describe demographic and medical characteristics, and between-group comparison of outcomes at baseline. There were no statistically significant between-group differences for demographic characteristics or outcomes measures at baseline.

**Table 1:** Baseline data table.

	pSMA group	Usual care group	P value
**Mean age in years (SD)**	55.89 (11.60)	60.11 (7.67)	0.376^†^
**Gender**			0.637*
- ***Male***	6	2	
- ***Female***	3	7	
**Ethnicity**			0.842*
- ***European***	3	3	
- ***Oceanic***	3	2	
- ***Asian***	3	4	
**Mean years diagnosed with T2DM (SD)**	16.67 (5.97)	14.11 (10.54)	0.536^†^
**Smoking status**			1.000^‡^
- ***Ex-smoker***	2	2	
- ***Never smoked***	7	7	
**Complications of T2DM**			
- ***Ischemic heart disease***	1	2	
- ***Stroke***	0	0	
- ***Peripheral vascular disease***	0	0	
- ***Foot ulcers***	0	1	
- ***Retinopathy***	0	1	
- ***Chronic renal failure***	0	0	
- ***Neuropathy***	1		
**Co-morbidities**			
- ***Non-alcoholic steatotic hepatitis***	0	1	
- ***Hypercholesterolemia***	3	5	
- ***Hypertension***	5	4	
- ***Hypothyroidism***	2	2	
- ***Depression***	0	2	
**On insulin therapy**	4	2	0.620^‡^

^†^T^2^ test; ^‡^Fisher’s exact test; Mann-Whitney U test *Pearson Chi square; SD, standard deviation.

**Table 2:** Between-group comparison of measures at baseline.

	pSMA group mean (SD)	Usual care group mean (SD)	P value
**Glycemic control and lipids**			
Hb1Ac-NGSP (%)	7.92 (1.09)	7.30 (0.44)	0.117^‡^
Hb1Ac-IFCC (mmol/mol)	62.89 (11.90)	56.25 (4.86)	0.117^‡^
Mean glucose (from CGMS) (mmol/L)	11.56 (4.06)	9.66 (2.01)	0.392^‡^
Time in range (from CGMS) (%)	40.89 (22.84)	60.71 (26.79)	0.132^†^
Triglycerides (mmol/L)	1.93 (0.63)	1.71 (1.15)	0.583^‡^
Total Cholesterol (mmol/L)	4.13 (0.61)	3.95 (0.63)	0.552^†^
**Anthropometric measures and blood pressure**			
Height (cm)	168.78 (5.17)	166.19 (12.32)	0.572^†^
Weight (kg)	96.67 (19.91)	81.67 (20.60)	0.136^†^
Waist circumference (cm)	112.39 (12.44)	102.50 (9.80)	0.091^†^
Hip circumference (cm)	114.67 (17.71)	108.63 (3.29)	0.945^‡^
BMI (kg/m^2^)	34.08 (7.86)	27.61 (2.99)	0.167^‡^
Waist/hip circumference ratio	0.99 (0.08)	0.94 (0.74)	0.259^†^
Systolic BP (mm/Hg)	139.11 (16.83)	133.25 (11.00)	0.416^†^
Diastolic BP (mm/Hg)	84.99 (7.15)	81.25 (4.65)	0.239^†^
**Beck Depression Inventory**	6.11 (4.83)	9.63 (10.13)	0.725^‡^
**State Anxiety Index**	33.11 (8.34)	33.86 (10.67)	0.877^†^
**EQ5D5L**			
- anxiety	1.44 (0.53)	1.63 (0.92)	1.000^‡^
- mobility	1.33 (0.50)	1.38 (0.74)	1.000^‡^
- pain discomfort	2.11 (0.93)	1.88 (0.84)	0.591^†^
- self-care	1.00 (0.00)	1.00 (0.00)	NA
- usual activity	1.56 (0.53)	1.50 (0.76)	0.819^‡^
- VAS	74.00 (16.96)	80.75 (15.66)	0.409^†^
**PAID**	23.06 (17.07)	31.88 (30.66)	0.468^†^
**PROMIS29**			
- pain intensity	2.33 (2.55)	2.00 (2.77)	0.682^‡^
- anxiety	2.11 (1.69)	3.25 (4.06)	0.935^‡^
- depression	1.67 (2.24)	2.38 (3.11)	0.904^‡^
- fatigue	4.00 (4.03)	4.88 (4.49)	0.678^†^
- pain interference	2.89 (3.59)	2.00 (3.89)	0.499^‡^
- physical function	1.78 (1.92)	1.38 (1.77)	0.636^‡^
- sleep	7.00 (1.12)	7.00 (1.15)	1.000^†^
- social	11.67 (5.17)	11.88 (3.64)	0.867^‡^

^†^T^2^ test; ^‡^Mann-Whitney U test.

HbA1c, glycated haemoglobin; NGSP, National Glycohemoglobin Standardization Program; IFCC, International Federation of Clinical Chemistry.

EQ5DL, EuroQol-5D; PROMIS-29, Patient-Reported Outcomes Information System questionnaire; PAID, Problem Areas in Diabetes scale; BMI, Body Mass Index; BP, blood pressure; VAS, Visual Analogue Scale.

### Primary Outcomes

#### Recruitment, Retention, and Adherence

Our recruitment resulted in an average of 26 enquiries and nine enrolments per month of active recruitment. The conversion rate from enquiry to enrolment was 37.5% (18/48) and from eligible to enrolled was 85.7% (18/21). From those that were excluded, 40% (12/30) did not meet the study’s inclusion criteria, 33.3% (10/30) were lost to follow up, 13.3% (4/30) declined to participate (no reason provided), 10% (3/30) were eligible for the study but were unable to attend the baseline visit and one participant declined to participate over safety concerns (see **Figure 1**). Of the 18 participants enrolled, 17 completed the 12-week intervention and outcome measures (94.4% retention). All of the participants in the intervention group completed at least 4 of 6 PSMAs.

#### Acceptability: Exit Questionnaire Data

Based on exit questionnaire data, most of the participants in the pSMA group found the education sessions helpful (8/9), enjoyed being able to talk to other people with diabetes (7/9), contributing to research (7/9), found the mindfulness sessions helpful (6/9), and enjoyed the extra attention toward their health and wellbeing (5/9) (**Table 1**). However, few participants reported finding the mindfulness home practice or workbooks helpful (2/9), or reported losing weight (2/9).

Some quotes around this question included: “*it was a great experience attending group sessions*” (ID3); “*being in the control group I did not get much out of the study, except the opportunity to wear a sensor*” (ID1); “*Nothing to me was detrimental. The experience helped me to better understand my diabetes through the course contents and interaction with the other participants*” (ID6). Participants reported for example “*It was great being able to share with others, and hearing others talk about their health and see the same things we go through*” (ID10), “*This is my first experience in SMA. I liked it and feel better than a one-on-one GP appointments*” (ID16).

When participants were asked what they did not enjoy about the trial, six from the pSMA group and four from the control group reported there was “*nothing*” that they did not enjoy. One participant said the SMAs were too far away from where he/she lived/worked, one did not enjoy the mindfulness practice, and one reported a technical error with a CGMS sensor. In the control group, one participant noted that the exit survey should have been tailored to the allocation groups (i.e., not asking control group participants the questions about PSMAs), and one noted that they could not access continuous glucose monitoring results in real time using a mobile application as advised, because the sensors that were used were not compatible with the Australian mobile application. Three participants from the control group reported that the “*did not learn anything new*”.

##### Shared Medical Appointments

There were six responses from the question about feedback on the SMA’s overall. All of the six responses were positive comments, e.g., “*very good*” (ID16), and “*excellent and should do more for these sessions*” (ID3).

The most important features of PSMAs according to participants were:

- The ability to share experiences and tips in a group discussion (6/9), including “*a better idea of the effect diabetes has on people [and] how they managed their diabetes and lived their lives*” (ID6);
- Expanded knowledge of diabetes from health professionals (5/9), which included education on results of tests, side effects of medications, a better understanding of diabetes overall, and increased confidence and awareness about diabetes;
- Sense of not being alone in experience of diabetes (5/9), including realizing that other people with diabetes were also struggling with their weight; and
- The style and approach of the facilitator and GP (friendly and non-judgmental, “*knowledgeable” (ID 18), “just made it easier to talk about things” (ID 10), “relaxed environment*” (ID 6) where participants felt free to ask questions) (5/9).

**Table 3** describes the additional feedback on pSMAs.

**Table 3:** Additional overall feedback about pSMAs from exit questionnaire.

Positive	N of responses	Other feedback/suggestions	
Continuous glucose monitoring experience was beneficial	2	Sessions could have been a bit longer	1 (ID11)
Enjoyed mindfulness meditation	2	Information provided in the last session about decisions to eat healthy/unhealthy food should have been presented in the first session as it was valuable information	1 (ID4)
Improved eating habits	1 (ID15)		
Learned that diabetes is not my fault	1 (ID4)		
Time and location was convenient	1 (ID8)		
“*they could be very effective with the right people”*	1 (ID10)		
Sessions could have been more frequent	1 (ID16)		

pSMAs, programmed Shared Medical Appointments.

Representative quotes include: “*Timing, duration and frequency was just right for me*” (ID16); “*was able to understanding how other where struggling/controlling their diabetes*” (ID3); “*being able to share and relate to others was very helpful*” (ID10); “*GP was knowledgeable and explained side effects of medications. In my experience doctors haven’t done this*” (ID18).

###### Facilitator and GP

We received eight responses for feedback on the facilitator and seven for the GP. All were positive with the facilitator rated from “*fantastic*” (ID16), to “*good*” (ID6). Participants described the facilitator as “*really good and knowledgeable*” (ID3), “*made us all feel relaxed*” (ID10), “*thoughtful*” (ID4). The characteristics of being friendly, approachable, and knowledgeable, were highly valued by the participants. Similarly, the GP was rated from “*good*” to “*the best person*” (ID3). ID4 noted “*Wow what a gentle caring GP*”.

###### Mindfulness

Six participants provided feedback on the mindfulness sessions. Four of these participants had positive feedback including “*exceptionally good*” (ID16) and “*very helpful and relaxing*” (ID10). ID4 wrote “*In walked this quite softly spoken woman and I thought what is this. Within 15 mins I was hooked. Relaxing and insightful*”. Two participants did not find the sessions useful.

Six participants from the pSMA group provided feedback on home mindfulness practice. Feedback was mixed. One participant noted that more assistance was needed (ID16). Another noted that, while mindfulness could be helpful, it was “*very difficult for busy people*” (ID10). Two participants found home practice helpful while another did not find it helpful.

###### Trial Procedures

**Table 4** describes the results on acceptability of trial procedures.

**Table 4:** Acceptability of trial procedures from the exit questionnaire.

Positive	N of responses	Other feedback/suggestions	N of responses
**Overall feedback on trial procedures**			
No change required	6 (2xSMA, 4xcontrol)	Should be expanded to a larger group	2 (1xSMA, 1x control)
		More advertisement is required to raise awareness about the trial	1 (ID 16)
No response			8
**Surveys**			
No problems noted	5	Too long	2
		Questions were too private and personal	1
No response			9
**Location of clinic visits**			
No problems noted	8		
On-site parking was helpful	1		
No response			8
**Glucose sensor**			
No problems noted	5	Better to have used Australian sensors which synchronized to an app for real-time monitoring	4
		Difficulty with sensor remaining in place	1
No response			7
**Pathology**			
No problems noted	7	Test results were wrongly recorded by pathology company	1
		“*a few things needed to be improved”* (no further information given)	1
No response			8
**Accelerometers**			
Positive or neutral (from “good” to “okay”)	5	A little inconvenient to wear	1
		Took a while to get used to	1
		Not helpful as could not access results in real time	1
No response			9

SMA, Shared Medical Appointment.

#### Qualitative Evaluation of Trial Feasibility and Acceptability

Seven out of the nine participants from the pSMA group contributed to the qualitative assessment of trial feasibility and acceptability (five males and two females). One focus group was held on the 2^nd^ of October 2019, attended by five participants, and two interviews were conducted over the telephone.

**Figure 2:**
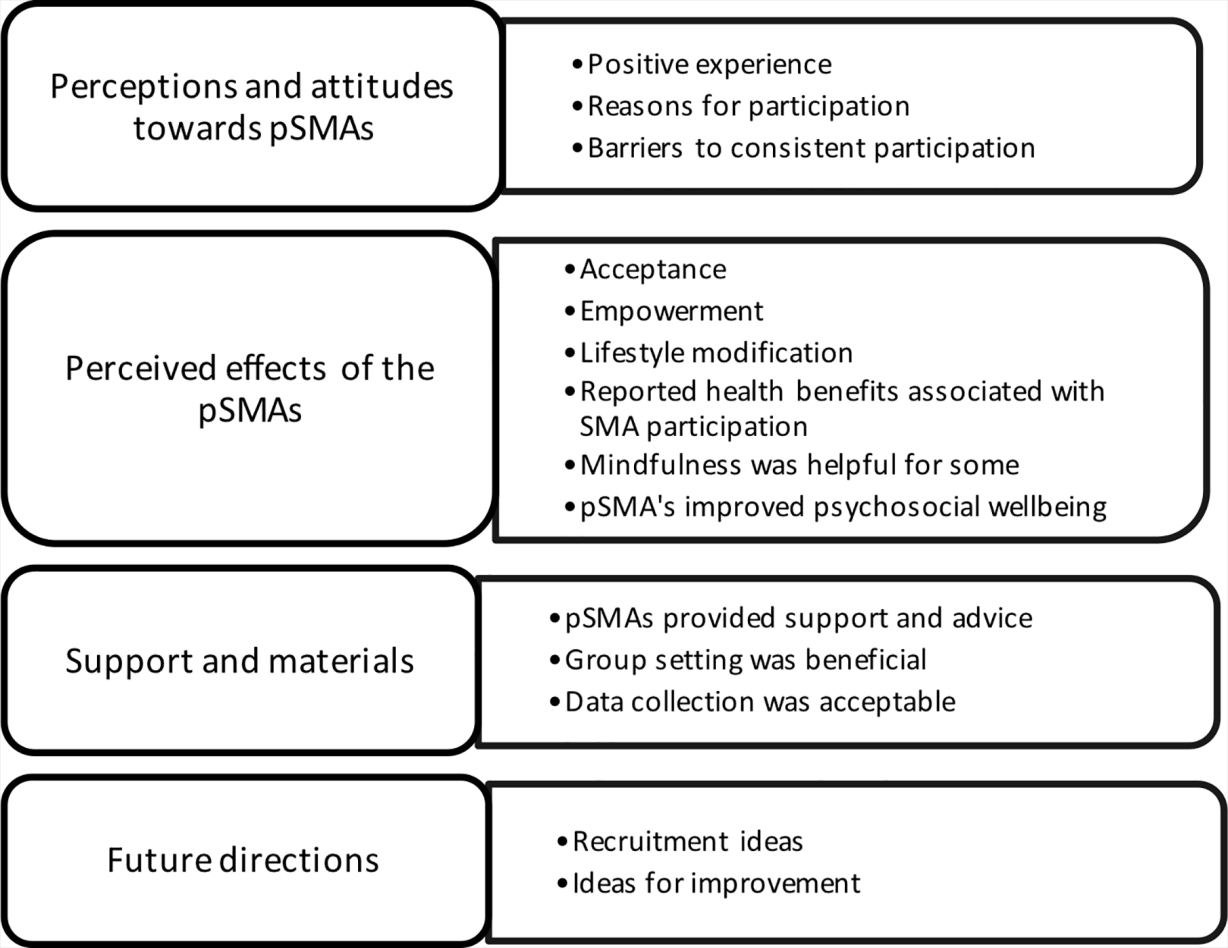
Themes and subthemes from qualitative evaluation. pSMAs, programmed Shared Medical Appointments.

Four central themes meaningful to the study were identified from the data: (1) perceptions and attitudes toward pSMAs, (2) perceived effects of the pSMAs, (3) support and materials, and (4) future directions (see **Figure 2**).

##### Theme 1: Perceptions and Attitudes Toward PSMAs

###### Positive Experience

Participants generally spoke positively about the program with many mentioning that they felt that it was “a very good project” (ID 6) and that the pSMAs were enjoyable to attend (excerpt 1.1a, b). Participants were generally satisfied with the frequency and duration of the pSMAs (excerpts 1.1c), as well as the time and location of them (excerpt 1.1d). Further, the longer duration of the pSMAs was also seen to be beneficial when compared to the shorter timeframe of individual medical visits (excerpt 1.1e, f). Participants were also prompted to discuss any negative impacts they may have experienced during the program. None of the participants reported having any negative or adverse events occur during the pSMAs, besides an adverse reaction to the glucose sensor mentioned below under *data collection* (excerpt 1.1g).

###### Reasons for Participation

The most commonly mentioned reasons for attending the pSMAs were to receive more information about the disease and treatment (excerpt 1.2a, b), and participants’ expectation to share experiences as well as learn from fellow patients with T2DM (excerpt 1.2c). Other reasons for attending the pSMAs included encouragement from family (excerpt 1.2d), and an interest in the mindfulness aspect of the program (excerpt 1.2e).

###### Barriers to Consistent Participation

Nearly all of the participants that were interviewed reported missing one out of the eight pSMAs in the study. Reasons listed for missing a pSMA included work commitments (excerpt 1.3a), a pre-booked holiday (excerpt 1.3b), and family reasons.

##### Theme 2: Perceived Effects of the pSMAs

###### Acceptance

Denial of the importance of diabetes, or even the diagnosis itself, was mentioned by participants with ID1 describing that they had “*tolerated being a diabetic and not accepted it*” (excerpt 2.1a). Some participants indicated that the stigma surrounding T2DM created feelings of “guilt” (ID 5) which inhibited their willingness to accept having the disease (excerpt 2.1b, c). However, these participants felt that attending the pSMAs helped to reduce the stigma and guilt associated with having T2DM, particularly due to the education they received surrounding its hereditary component. This, in turn, led to a sense of acceptance for those who had previously struggled to come to terms with having T2DM (excerpt 2.1c, d).

###### Empowerment

All of the participants reported an increase in diabetes knowledge and awareness since attending the pSMAs. Some participants felt that they were now better equipped to manage their diabetes and improve their health (excerpt 2.2a, b). Further, many participants noted feeling a sense of empowerment from the education they received during the pSMAs, with ID1 saying that the sessions were “*all about empowering you*” (excerpt 2.2c, d). Several participants utilized the education they received during the pSMAs, and applied it in their everyday lives as listed below under *lifestyle modifications*. Even those that had yet to make any lifestyle modifications felt that they were now more inclined to try and make changes to improve their health and diabetes (excerpt 2.2e)

###### Lifestyle Modification

Participants reported applying the education they received on diabetes, diet, and health during the pSMAs to their lifestyles. Many of the participants reported an improved awareness of their diets and the food that they were consuming (2.3a). This was indicated *via* changes made to their diet including reducing consumption of refined carbohydrates such as rice, bread and pasta (excerpt 2.3b), eating smaller portion sizes (excerpt 2.3c), and making a conscious effort to swap junk food for healthier options (excerpt 2.3d). Other lifestyle modifications included making an increased commitment to looking after health *via* regular exercise and the practice of mindfulness made by one participant (excerpt 2.3e). Another participant mentioned focusing on reducing stress from their life after learning of the impact it can have on health in one of the pSMAs (excerpt 2.3f). Several of the participants had not yet made any lifestyle modifications associated with their participation in the pSMAs (excerpt 2.3g). Reasons for this included issues in their personal life (excerpt 2.3h)and difficulty in gaining motivation to make healthy lifestyle changes (excerpt 2.3i). One participant also noted that it was more difficult for them to reduce their carbohydrate intake due to rice being the staple food in their Asian culture (excerpt 2.3j).

###### Reported Health Benefits Associated With SMA Participation

Several participants reported noticing benefits to their health since participating in the pSMAs. Two of the participants reported experiencing weight loss (excerpt 2.4a), crediting the education they received surrounding diet and food choices and consequent lifestyle changes, as the reasons for this weight loss. One participant noted a decrease in their HbA1c levels at the end of the pSMAs (excerpt 2.4b), whilst another reported experiencing an increase in energy levels as a result of their participation in the pSMAs (excerpt 2.4c). An increase in joint and muscle flexibility was also mentioned as a health benefit experienced by one participant (excerpt 2.4d). A few of the participants stated that they were yet to experience any health benefits associated with their participation in the pSMAs (excerpt 2.4e). These participants were typically the ones that had not yet made any lifestyle modifications, as mentioned above.

###### Mindfulness was Helpful for Some

pSMA participants enjoyed practicing mindfulness and found it beneficial for various reasons. Several participants found that practicing mindfulness helped to improve the decision-making process behind one’s dietary choices, which in turn, helped reduce mindless snacking and the consumption of “junk” food (excerpt 2.5a, b). Others found that practicing mindfulness improved sleep and enhanced relaxation and reduced stress (excerpt 2.5c–e). In regard to the practice of mindfulness at home, approximately half of the participants reported doing so whilst the other half stated that they had not yet done so (excerpt 2.5f, g). There were also a few participants that believed that mindfulness did not have any impact on their health or wellbeing, specifically stating that “mindfulness didn’t work” (excerpt 2.5h).

###### pSMAs Improved Psychosocial Wellbeing

Participating in pSMAs appeared to positively impact on the mood of many of the participants (excerpt 2.6a). Many of the participant responses indicated that they enjoyed attending the fortnightly pSMAs. Some of the participants also mentioned the mindfulness sessions included in the pSMAs as a contributing factor to their enhanced mood and decreased stress levels.

##### Theme 3: Support and Materials

###### pSMAs Provided Support and Advice

Content provided during the education sessions was found to be useful, beneficial, and easy to understand (excerpt 3.1a). Participants generally found that the information handouts they received throughout the pSMAs were useful and straightforward, and a few also mentioned using the notebook/diary provided to take notes during the session (excerpt 3.1b, c). Participant responses also indicated that they were pleased with the support and advice provided throughout the pSMAs, with many stating that they found the facilitators to be helpful and understanding (excerpt 3.1d). Further, importance was placed on the ability of the facilitators to create a comfortable environment and encourage the group to ask questions and share their experiences without feeling insignificant (excerpt 3.1e, f). One participant described being initially shy to talk in a group setting but once the pSMAs began they felt comfortable to engage and to ask questions (excerpt 3.1g). Overall, none of the participants reported any issues with the level of support and advice they were provided with throughout the pSMAs.

###### Group Setting Was Beneficial

The group setting was seen as beneficial by all participants, particularly because they reported finding it useful to hear about others experiences with diabetes as well as to share their own (excerpt 3.2a–c). Further, participants found it easy to learn from others in this group environment, mainly in regard to the management of diabetes (excerpt 3.2d, e). Participants also noted feeling that their different views were valued (excerpt 3.2f), and stated that they enjoyed bonding with other people in the “*same boat*” (ID 1) as them (excerpt 3.2g). Some participants mentioned an initial concern regarding how comfortable and willing everyone would be to share their experiences in front of strangers, but one participant found that it “*very quickly became comfortable*” (excerpt 3.2h, ID 5). It was also agreed by many of the participants that it was more favorable to attend the pSMAs with strangers as opposed to people you know, such as relatives and friends (excerpt 3.2i). One participant felt that being with strangers results in a “*lack of inhibition and embarrassment*” (ID 2), making it easier to share experiences and ask questions.

###### Data Collection Was Acceptable

The participants generally felt that the data collection process was easy and ran smoothly (excerpt 3.3a). Participants felt that it was useful to find out their measurements, including weight and blood sugar levels, and it was considered a good progress checkpoint by some participants (excerpt 3.3b, c). There were no major issues with the data collection process; however, one participant had an adverse reaction to the glucose sensor resulting in blisters and itchiness (excerpt 3.3d). Another participant mentioned receiving incorrect instructions in regard to the use of the accelerometer. One participant also felt that there were “too many questions” (ID 6) included in the data collection process (excerpt 3.3e).

##### Theme 4: Future Directions

###### Recruitment Ideas

Promotion of pSMAs *via* GPs, endocrinologists, and the National Diabetes Services Scheme (NDSS), as well as *via* word of mouth, were all considered to be important for future SMA recruitment (excerpt 4.1a, b). Further, many of the participants stated that they would recommend pSMAs to other T2DM patients (excerpt 4.1c). The challenges to recruiting others to attend SMAs discussed by participants included potential feelings of shyness inhibiting one’s willingness to share their experiences in a group setting (excerpt 4.1d) as well as a lack of time and willingness to commit (excerpt 4.1e).

###### Ideas for Improvement

Participants were prompted to discuss aspects of the pSMAs that could be improved to enhance their effectiveness. Two participants mentioned that they would like the information delivered during the pSMAs to be provided digitally *via* a PDF rather than in hard copy, with one suggesting the creation of an app that contains this information (excerpt 4.2a, b). With regards to long-term use of pSMAs, participant responses indicated that they were satisfied with attending over the 8-week period but would not want to attend for longer periods of time. However, some of the participants mentioned continued contact and updates would be useful (excerpt 4.2c).

### Secondary Outcomes

#### Anthropometric Measures

There were no statistically significant differences between groups at post intervention for weight, waist circumference, hip circumference, BMI, WHR, or blood pressure (see **Table 5**).

**Table 5:** Anthropometric measures and blood pressure at baseline and 12 weeks.

	pSMA group mean baseline (SD)	pSMA mean post intervention (SD)	Usual care group mean baseline (SD)	Usual care group mean post intervention (SD)	P value
**Weight (kg)**	96.67 (19.91)	95. 78 (19.84)	76.63 (14.96)	75.75 (14.98)	0.993^†^
**Waist circumference (cm)**	112.39 (12.44)	111.86 (15.41)	102.50 (9.80)	103.75 (8.24)	0.132^‡^
**Hip** **circumference (cm)**	114.67 (17.71)	114.20 (17.49)	108.63 (3.29)	107.38 (3.70)	0.510^†^
**BMI (kg/m^2^)**	34.08 (7.86)	33.78 (8.02)	27.61 (2.99)	27.23 (2.83)	0.885^†^
**Waist/hip circumference ratio**	0.99 (0.08)	0.98 (0.66)	0.94 (0.74)	0.97 (0.06)	0.183^†^
**Systolic blood pressure (mm/Hg)**	139.11 (16.83)	137.67 (14.34)	133.25 (11.00)	131.50 (15.79)	0.962^†^
**Diastolic blood pressure (mm/Hg)**	84.99 (7.15)	83.00 (4.50)	81.25 (4.65)	79.88 (8.90)	0.880^†^

^†^T^2^ test; ^‡^Mann-Whitney U test.

BMI, Body Mass Index.

#### Glycemic Control and Lipids

There was a trend to improvement in glycemic control (glycated hemoglobin, continuous glucose levels, and time in range) in the pSMA group and a deterioration in the usual care group; however, these differences were not statistically significant. There was a statistically significant difference between groups for change in total cholesterol levels, which decreased by 6.5% in the pSMA group and increased by 5% in the usual care group (see **Table 6**).

**Table 6:** Glycemic control and lipids at baseline and 12 weeks.

	pSMA group mean baseline (SD)	pSMA group mean post intervention (SD)	Usual care group mean baseline (SD)	Usual care group mean post intervention (SD)	P value
**Hb1Ac-NGSP (%)**	7.92 (1.09)	7.81 (0.80)	7.30 (0.44)	7.5 (1.03)	0.336^†^
**Hb1Ac-IFCC (mmol/mol)**	62.89 (11.90) (11.90) (11.90)	61.89 (8.67)	56.25 (4.86)	58.50 (11.41)	0.356^†^
**Mean glucose (from CGMS) (mmol/L)**	11.56 (4.06)	10.52 (1.95)	9.66 (2.01)	10.37 (2.49)	0.555^‡^
**Time in range (from CGMS) (%)**	40.89 (22.84)	49.60 (24.23)	60.71 (26.79)	55.14 (30.85)	0.296^†^
**Triglycerides (mmol/L)**	1.93 (0.63)	2.13 (1.06)	1.71 (1.15)	2.01 (1.03)	0.583^‡^
**Total Cholesterol (mmol/L)**	4.13 (0.61)	3.86 (0.66)	3.95 (0.63)	4.15 (0.73)	**0.025^‡^**

^†^T^2^ test; ^‡^Mann-Whitney U test; NGSP, National Glycohemoglobin Standardization program; IFCC, International Federation of Clinical Chemistry; CGMS, Continuous Glucose Monitoring System. Bold figures represent statistically significant differences.

#### Psychological Outcomes and Quality of Life

**Table 7** presents the findings for psychological outcomes and quality of life. Psychological outcomes improved in the pSMA group compared to little change in the usual care group; however, these changes were not statistically significant. This included a 36.3% reduction in the Beck Depression Inventory and 41.5% improvement in the PAID score in the pSMA group.

**Table 7:** Psychological outcomes and quality of life at baseline and 12 weeks.

	pSMA group mean baseline (SD)	pSMA group mean post intervention (SD)	Usual care group mean baseline (SD)	Usual care group mean post intervention (SD)	P value
**BDI**	6.11 (4.83)	3. 89 (3.30)	9.63 (10.13)	9.88 (11.45)	0.832^‡^
**SAI**	33.11 (8.34)	30.11 (8.09)	33.86 (10.67)	36.29 (15.27)	0.281^‡^
**EQ5D5L**					
**- anxiety**	1.44 (0.53)	1.44 (0.53)	1.63 (0.92)	1.75 (1.67)	0.817^‡^
**- mobility**	1.33 (0.50)	1.22 (0.44)	1.38 (0.74)	1.38 (0.74)	0.939^‡^
**- pain discomfort**	2.11 (0.93)	1.67 (0.50)	1.88 (0.84)	2.00 (0.93)	0.187^‡^
**- self-care**	1.00 (0.00)	1.00 (0.00)	1.00 (0.00)	1.00 (0.00)	NA
**- usual activity**	1.56 (0.53)	1.22 (0.44)	1.50 (0.76)	1.13 (0.35)	1.000^‡^
**- VAS**	74.00 (16.96)	75.44 (14.14)	80.75 (15.66)	75.88 (23.66)	0.646^‡^
**PAID**	23.06 (17.07)	13.47 (7.75)	31.88 (30.66)	30.16 (34.03)	0.129^†^
**PROMIS29**					
**- pain intensity**	2.33 (2.55)	2.11 (2.37)	2.00 (2.77)	2.38 (2.93)	**0.034^‡^**
**- anxiety**	2.11 (1.69)	1.67 (1.58)	3.25 (4.06)	3.13 (4.39)	0.601^‡^
**- depression**	1.67 (2.24)	1.00 (1.58)	2.38 (3.11)	3.75 (4.23)	0.054^‡^
**- fatigue**	4.00 (4.03)	3.56 (2.92)	4.88 (4.49)	5.63 (4.81)	0.252^†^
**- pain interference**	2.89 (3.59)	2.67 (3.57)	2.00 (3.89)	2.63 (5.68)	0.388^‡^
**- physical function**	1.78 (1.92)	1.00 (1.12)	1.38 (1.77)	1.88 (2.90)	0.129^‡^
**- sleep**	7.00 (1.12)	6.89 (0.78)	7.00 (1.15)	7.75 (0.71)	0.217^†^
**- social**	11.67 (5.17)	11.67 (4.72)	11.88 (3.64)	11.75 (4.56)	0.938^†^

^†^T^2^ test; ^‡^Mann-Whitney U test; BDI, Beck Depression Inventory; SAI, State Anxiety Index; EQ5D5L, EuroQol 5 D; PROMIS-29, Patient-Reported outcomes Information System questionnaire; PAID, Problem Areas in Diabetes Scale. Bold figures indicate statistically significant differences.

Fatigue, pain, and physical function improved in the PROMIS 29 domains in the pSMA group but only the change in pain intensity was statistically significant; however, the mean score for this suggested low levels of pain intensity at baseline.

#### Diet and Physical Activity Changes

**Table 8** describes the findings for diet and physical activity changes. There was a significant difference between groups at baseline for weekly takeaway intake (*p* = 0.0451) with the intervention group consuming a greater number of takeaway meals than the control group. There was no difference between groups at post intervention for physical activity levels and dietary habits.

**Table 8:** Diet and physical activity habits at baseline and 12 weeks.

	pSMA group mean baseline (SD)	pSMA group mean post intervention (SD)	Usual care group mean baseline (SD)	Usual care group mean post intervention (SD)	P value
**Sedentary behavior per day (min/h per day of wear time)**	859.57/14.3 (134.26/8.59)	868.60/14.47 (131.21/2.19)	778.48/12.97 (234.25/3.90)	739.13/12.32 (208.08/3.47)	0.203^†^
**Light PA (min/h per day of wear time)**	168.36/2.806 (51.74)	153.79/2.56 (44.48)	193.37/3.22 (71.85)	197.95/3.30 (63.04)	0.521^†^
**Moderate PA (min per day of wear time)**	34.26 (19.11)	37.37 (26.69)	39.13 (16.54)	39.01 (21.87)	0.308^†^
**Vigorous PA (min per day of wear time)**	0.48 (0.71)	0.40 (0.76)	0.77 (0.86)	0.79 (1.31)	0.876^‡^
**Average MVPA Per Day (min)**	34.74 (19.50)	37.77 (27.10)	39.89 (16.65)	39.81 (21.65)	0.365^†^
**Calendar days with wear time**	6.71 (0.76)	6.86 (1.07)	6.40 (0.55)	6.00 (0.71)	0.235^‡^
**Total wear Time**	7,205.60 (1,457.93)	7363.44 (1785.76)	6,608.25 (1,716.10)	6,411.43 (1,720.19)	0.180^†^
**Daily step count**	6,165.66 (2,579.86)	6,476.41 (3,419.28)	8,498.48 (2,329.73)	7,201.38 (1,862.91)	0.699^†^
% sedentary*	80.60 (5.39)	81.23 (4.02)	74.55 (12.31)	74.07 (10.42)	0.670^†^
**% light PA**	16.42 (4.45)	15.30 (3.21)	20.81 (11.20)	21.68 (10.05)	0.419^†^
**% moderate PA**	2.97 (1.54)	3.42 (2.41)	4.54 (1.25)	4.13 (1.23)	0.457^†^
**% vigorous PA**	0.02 (0.02)	0.41 (0.07)	0.09 (0.10)	0.11 (0.18)	0.935^‡^
**% MVPA**	2.99 (1.55)	3.46 (2.44)	4.64 (1.26)	4.24 (1.17)	0.463^†^
**Daily serves of vegetables**	2.06 (1.57)	1.75 (1.14)	1.52 (1.04)	1.48 (0.51)	0.799^‡^
**Daily serves of fruit**	1.06 (0.39)	1.13 (0.47)	1.37 (0.85)	1.52 (0.62)	0.974^†^
**Weekly serves of takeaways**	5.78 (4.74)	4.78 (3.03)	1.89 (2.52)	1.625 (1.30)	1.00^‡^

T^2^ test; ^‡^Wilcoxon rank-sum (Mann-Whitney); *percentages are reported as percentage of wear time.

Calendar days with wear time at Baseline: 0.222^‡^.

Calendar days with wear time at Post-intervention: 0.151^†^.

We were only able to calculate mean minutes per day and days per week of meditation as many of the logs were incomplete or reported only according to a brief estimate of time spent meditating. Minutes per day, days per week of meditation, and minutes per week of meditation were not normally distributed in the usual care group due to an outlier who practiced meditation for an hour a day 7 days a week, and so a non-parametric test was used. There was no difference between groups for minutes per day (*p* = 0.360), days per week (*p* = 0.479), or minutes per week (*p* = 0.626) spent meditating over the intervention period. When we removed the outlier from the usual care group, there was still no difference between groups for minutes per day (*p* = 0.629), days per week (*p* = 0.289), or minutes per week (*p* = 1.00). **Table 9** provides details of mean time spent meditating in both groups, with one outlier removed from the usual care group.

**Table 9:** Time spent meditating throughout intervention period.

	pSMA group (n = 9)	Usual care group (n = 7)
**Mean minutes per day (SD)**	2.14 (2.50)	10.24 (20.33)
**Mean days per week (SD)**	3.67 (3.54)	1.07 (1.43)
**Mean minutes per week (SD)**	14.11 (17.12)	13.10 (20.77)

#### Harms

Eighteen adverse events (AE) were reported. Of these, five were determined to be definitely caused by participation in the trial (but not due to the intervention itself), and 13 were deemed to not be related to the intervention. There were three reports of mild pain around the CGM sensor, which were short-lived and completed resolved within 10 min. There was one report of itch and redness around the sensor, which was mild and partially resolved at the time of follow-up. There was one report of a mild bruise around the phlebotomy site. The other AEs (which were deemed not related to the intervention) were back pain, blurred vision, cough, headache, high glucose level due to a urinary tract infection, elective surgery for an abdominal hernia, a toe injury (which did not occur while attending to trial activities), loose teeth, migraine, tightness of the chest, a tooth extraction, an upper respiratory tract infection, and visual disturbance. Ten AEs occurred in the pSMA group (including two definitely related AEs) and eight in the usual care group (including three definitely related AEs). One serious AE was reported (elective surgery as described above), which was not related to the intervention.

## Discussion

In our mixed-methods study of pSMAs with mindfulness for people with T2DM within primary care, we demonstrated high levels of acceptability and feasibility of implementation of both the intervention and follow-up trial. Notably, the majority of participants from the pSMA group and half of the participants in the control group reported that there was nothing that they did not enjoy about taking part in the study. Qualitative evaluation demonstrated that pSMA participants found the program to be positive overall, with multiple benefits such as increased acceptance of their diagnosis, feeling empowered, making changes with their lifestyle, and enjoying the opportunity to meet people who were in the “same boat” as them. Our findings are consistent with previous studies on SMAs for people with T2DM with patient satisfaction generally being high, diet and physical activity habits improving, and patients being able to achieve or almost achieve their self-prescribed goals (10).

SMAs have been promoted as an effective way for clinicians to deliver care to multiple patients with the same clinical problem, therefore, increasing provider satisfaction (35–38). The impact on people with T2DM is multifactorial. The most important features of the SMAs, according to our participants, were the ability to share experiences in a group setting, an expanded knowledge of diabetes, a sense of not being alone, and the style and approach of the facilitator and GP.

The provision of additional support and advice was an important component of our pSMAs, according to participants. The design of SMAs allows for each participant to listen and gain from questions and answers during others’ medical consultations. This may have led to the positive changes in lifestyle habits that were described by some participants. This is supported by the objective increase in minutes and percentage of wear time spent in moderate to vigorous physical activity (MVPA) noted in the pSMA group, whereas there was no change in the control group. This difference equates to an additional 3 min of MVPA per day or 21 additional min per week (an increase of 8.6%). Similarly, other studies have reported improvements in patient engagement after attending SMAs, such as being able to set and achieve measurable goals and improvement in knowledge, self-efficacy and self-management (39). However, no difference between groups was observed for diet changes in our study, although we did find a statistically significant difference between groups for total cholesterol.

The group setting was seen as beneficial for several reasons. Participants described the benefits of being able to learn from each others’ experiences. The process of sharing one’s experiences and tips on management of a chronic disease may increase self-efficacy. It has been described that peer support for people with T2DM improves clinical and psychological outcomes particularly in minority populations, and may be associated with improvements in lifestyle habits, self-care, and self-efficacy (40–42).

Participants in the pSMA group described the positive impact of the program on their mental health. This included the ability to finally accept their diagnosis, the feeling of not being alone in their experience of T2DM, and being able to finally overcome guilt and stigma. This was reflected in the improvement in depressive symptoms and diabetes-related distress in the pSMA group, although the average score for depression was in the normal range and it is not clear if these differences are clinically relevant.

The process of overcoming guilt and stigma not only benefited mental health in our study; it was described by participants as empowering. Patient empowerment is defined as “*a process through which people gain control over decisions and actions affecting health*” and has been shown to improve self-management skills in people with T2DM, and have positive impacts on clinical, lifestyle and psychosocial outcomes, including diabetes complications (43). Empowerment requires not only the acquisition of skills and knowledge, but also a psychologically safe environment that facilitates self-reflection and self-awareness. People with T2DM commonly experience feelings of guilt and shame and experience stigma, which may become barriers toward the management of T2DM such as with initiating insulin or making lifestyle changes (44–46). These feelings, as well as fear of being judged by medical doctors, may act as a barrier to effective communication within a consultation (47). It was clear that participants felt a strong rapport with, and benefited from, the therapeutic relationship with the chosen facilitator and GP. The qualities of being knowledgeable, friendly and supportive were perceived highly, and led to participants feeling confident in asking questions and feeling cared for. Likewise, other studies have highlighted the importance of establishing trusting and nonjudgmental relationships within the patient-clinician dyad for people with T2DM in order to facilitate self-efficacy and improved clinical outcomes (48).

The combination of provision of knowledge in the educational component of the pSMAs, the ability to share knowledge with peers, and the therapeutic alliance with health providers in a safe environment may have facilitated the trends toward improvement in some clinical outcomes in the pSMA group. Although the differences in glycemic control were not statistically significant between groups, we report HbA1c improvement of 0.11% in the pSMA group and worsening by 0.2% in the control group. Similarly, mean glucose from CGMS decreased and time in range increased in the SMA group whereas the opposite was observed in the control group. This is consistent with findings from other studies reporting lower HbA1c (10), however, remains to be confirmed in a fully-powered RCT. However, there was little change in anthropometric measurements or blood pressure.

There was a mixed response to the mindfulness component with some participants providing positive feedback on the guided mindfulness sessions while others reporting they did not find the sessions useful. Although systematic reviews on mindfulness meditation in the general population report improvements in eating behaviors and physical activity (11–16), the evidence in T2DM is mixed. Trials that used Mindfulness-Based Stress Reduction, an intensive 8-week program pioneered by Jon Kabat-Zinn, have reported improvements in glycemic control (49) and psychological outcomes (49, 50) with an improvement in microalbuminuria at one year which was not sustained at two and three year follow-up (51). Other mindfulness-based interventions, such as mindful self-compassion, mindfulness-based cognitive therapy, and Acceptance and Commitment Therapy, described reductions in depression (52, 53) diabetes-related distress (52), glycemic control (52, 54), and better diabetes self-care (54). However, a program of mindful eating was similar to diabetes self-management education for improvements in depression, nutrition and eating-related self-efficacy, and cognitive control (55). It is not clear if mindfulness contributed to improvements in psychological outcomes, lifestyle habits and glycemic control in our study, although some participants did describe mindfulness helping them with making healthier food choices (for example, reducing mindless snacking) and reducing stress. Reductions in catecholamines have been described after the practice of mindfulness (51). A clinical trial comparing SMAs with mindfulness to SMAs alone is required to evaluate the impact of mindfulness on T2DM outcomes. Additionally, participants reported needing some assistance or guidance with home practice, and a more structured approach to home mindfulness practice may be required. Last, a limitation of this study design is the lack of an active control comparator. In order for a follow-up trial to be methodologically rigorous and add to the body of evidence on mindfulness, an active control should be utilized (56–58).

Recruitment in this study was highly feasible with 18 participants enrolled within two months. Unfortunately, we did not collect information on how participants found out about our study; however, we note that the majority of enquiries were made after a Diabetes NSW newsletter was emailed to members. The conversion rate from enquiry to enrolment was relatively high at 34.6%. This reflects our broad inclusion criteria. Our retention rate was excellent with only one participant withdrawing (from the control group). These findings demonstrate the feasibility of a follow-up fully-powered RCT within primary care. Additionally, our mixed methods evaluation of the acceptability of trial procedures demonstrates that participants were mostly satisfied with trial procedures. The only issues raised were relatively minor such as technical problems with the accelerometer or CGMS monitor.

Modifications for a follow-up trial, based on the feedback from our participants, should include support materials being delivered electronically, more support and guidance with home mindfulness practice (for example, direction and reminders to practice at a particular time of day such as before bed), and use of Australian CGMS sensors which would allow for real-time monitoring using a mobile app, reduction in the number of PROMs to be collected, and presenting information about diet early on in the program. Additionally, a waitlist control design would be ideal to maintain engagement within the control group. To evaluate the effect of SMAs + mindfulness vs. SMAs alone or waitlist control, a three-armed trial is warranted. Further, the personal qualities of the GP and facilitator are of great importance; these health professionals should be non-judgmental, approachable, supportive, and create trust within the therapeutic relationship.

Strengths of this study include its mixed-method design which allowed us to explore, in a profound way, the experiences of the participants in the pSMA group and how the pSMAs impacted on their health and wellbeing as well as their views on the reasons why pSMAs were helpful. We added a novel component, mindfulness, in order to explore the relationship between this technique and the ability to make decisions about healthy lifestyle habits and the impact on psychological health. We utilized objective and reliable measures of physical activity, collected outcomes on mental health and diabetes-related distress, and used continuous glucose monitoring as an additional measure of glycemic control. Limitations are the small sample size, including the sample size for the qualitative evaluation, and the relatively short duration of treatment of 12 weeks. Due to the pSMAs being conducted in English, we could not enrol people who could not understand or converse in English, therefore our findings are unable to be generalized to non-English speaking populations. Despite this, there was evidence of cultural diversity within our sample, with about a third of participants in both groups being of Asian origin, and a quarter to a third being of Oceanic peoples’ origin. Moreover, scaling up this intervention means that the same GP and facilitator may not be able to provide the intervention to all participants, and it is unknown what the impact of different personalities would be on the overall experience of pSMAs. This has implications on training and monitoring of the fidelity of the intervention in a future trial. Additionally, we did not collect data on provider satisfaction, nor on whether control group participants used any additional services such as the GetHealthy NSW coaching service that was suggested to them, or the meditation app. Future modifications may also include anthropometric measurements using bioimpedance to evaluate changes in fat free mass as well as total body weight, which has a greater impact on clinical outcomes, and incorporating health economic evaluation.

## Conclusion

Our program of pSMAs with mindfulness within primary care was acceptable and generally enjoyable for participants and led to improvements in diabetes knowledge, lipids, and psychological health. It is likely that these benefits were mediated by multiple factors such as the group setting (with peer support and realization that one is not alone in their experience of T2DM), improved knowledge and support facilitating improved lifestyle behaviors, and a strong therapeutic relationship with the facilitator and GP resulting in improved engagement and self-efficacy. Therefore, pSMAs may represent a cost-effective and time-efficient enhancement to T2DM management within primary care. Mindfulness may also have played a role in helping improve dietary choices and reducing stress for some participants. Our mixed methods study demonstrates feasibility of both the intervention and a follow-up fully-powered RCT to confirm or refute these benefits.

## Data Availability Statement

The raw data supporting the conclusions of this article will be made available by the authors, without undue reservation. The full protocol is available from corresponding authors upon reasonable request.

## Ethics Statement

The studies involving human participants were reviewed and approved by Western Sydney University Human Research Ethics Committee. The patients/participants provided their written informed consent to participate in this study.

## Author Contributions

CE conceived of the study and obtained funding. CE, DC, BC, GE, JS, SG, MM, CF, RW, FM, KM and GD contributed to the design of the study. NA, AB, MA-D, JH, KM, and SB contributed to data collection. All authors contributed to the article and approved the submitted version.

## Funding

This research was funded by a Women’s Research Fellowship grant from Western Sydney University, 2019.

## Conflict of Interest

As a medical research institute, NICM Health Research Institute receives research grants and donations from foundations, universities, government agencies, individuals and industry. Sponsors and donors provide untied and tied funding for work to advance the vision and mission of the Institute. GE and JS are voluntary Board members of the Australasian Society for Lifestyle Medicine, a member organisation which provides training on Shared Medical Appointments. GE and JS do not receive income from the training.

The remaining authors declare that the research was conducted in the absence of any commercial or financial relationships that could be construed as a potential conflict of interest.
