# Supplementary material for: Shared Medical Appointments and Mindfulness for Type 2 Diabetes—A Mixed-Methods Feasibility Study
Source: Front Endocrinol (Lausanne). 2020 Oct 6;11:570777. doi: 10.3389/fendo.2020.570777 (PMC7573307; doi:10.3389/fendo.2020.570777)
Supplement: Supplementary file 1 [file Table_1.docx]

**Shared Medical Appointments**

**Education Sessions – Diabetes NSW & ACT**

**Time per session:** 30 minutes

**Scope of the education session**

- Delivered by a credentialled diabetes educator
- Where possible the education session is linked to the topics discussed during the Shared Medical Appointment (SMA) or is the chosen of by the participants.
- Provide information and education to assist with self-management.
- Each topic will have learning objectives, suit different learning styles; visual (use of props) and kinaesthetic (touch/handle props), as well as auditory (delivery of the session without the use of a PowerPoint presentation).
- Provide details of where to go for further support such as NDSS services such as the Helpline and local programs.
- Encourage regular visits or reconnecting with their doctor and/or primary health care team.
- The program will be developed to encompass the **choice theory**; focusing on what the person wants to change based on their individual needs, delivered using positive health literacy language and tone, providing content that is user-centred.

**What support is available and will be promoted?**

- **Information book** - After registering with the National Diabetes Services Scheme (NDSS), the person will receive a registration card and an information book **within 10 business days**. The book is designed to give general information about diabetes, NDSS services and products.
- **National NDSS information sheets** on a large variety of topics are available. These can be downloaded or ordered through the diabetes organisations or the NDSS website.
- **NDSS Multicultural Portal**: provides a broad range of translated diabetes resources, including factsheets and brochures, about managing diabetes in a wide variety of languages.
- **National NDSS Diabetes Helpline:** a phone service staffed by a variety of health professionals, such as diabetes educators and dietitians, who can answer questions and provide practical, up-to-date information. The Helpline operates during 8:30am to 5:00pm Monday to Friday and from 9:00am to 12:00pm on Saturdays and national public holidays.

### **NDSS Education programs:** held locally and free to attend if registered with the NDSS, include for example:

- - **Carb Smart**: covering healthy eating, how carbohydrates impact blood glucose levels and how to choose good quality carbohydrates.
  - **Foot Smart**: covering daily foot care and how to avoid foot complications
  - **Med Smart**: covering what kind of medications the person uses, what they do and what the side effects could be.
  - **Shop Smart**: covering healthy eating and how to read food labels.
  - **Ready Set Go, Let’s Move**: which assist the person in taking the first steps to making physical activity a part of their lifestyle.
  - **Beat it Gym program:** 8 week (2 one-hour sessions per week) physical activity and lifestyle program, tailored exercises by accredited exercise physiologist in a group setting.
  - **Living with insulin:** workshop for people changing or have changed insulin injections, to learn more about how insulin works, blood glucose monitoring and hypo/hyperglycaemia.

**Sessions and learning objectives**

- Each session will close where to go for more support, and promotion of other local education options

| **TOPIC** | **LEARNING OBJECTIVES** | **VISUAL AIDS & activities** | **RESOURCES** |
| --- | --- | --- | --- |
| **What makes your blood glucose level go up or come down** | - To understand the action of insulin - To understand the effect of food, exercise, stress, illness and medication including steroids on the blood glucose level - To identify their target BGL range. - To recall the action to take if the blood glucose level is not in the target range. | **Activity:** using the diagram below look at the reasons why BGLs vary, the causes of diabetes and what effects the blood glucose level.  Group discussion and problem solving  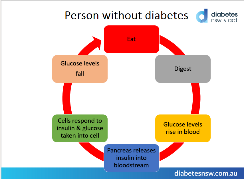  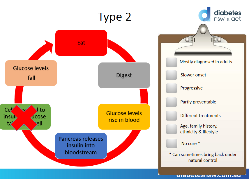 | NDSS Fact sheets:   - Type 2 diabetes |
| **Practical tip for monitoring your blood glucose levels** | - To understand when to check their blood glucose level - To recall how to check blood glucose level - To understand how to access testing strips, lancets and sharps - To understand the services provided by the NDSS | **Activity:** People will bring their meters  Discussion on how to use the equipment, check the strips are in-date, lancet usage (frequency before changing).  Everyone will check their meter with control solution.  People who are not checking their BGL will be provided with a meter and instruction. Meters will be replaced if an older model. | NDSS Fact sheets:   - Blood glucose monitoring   Supplies   - Spare meters to give away - Sharps containers to give away - Control solution |
| **Annual Cycle of Care** | - To understand the role of the Annual Cycle of Care in self-managing their diabetes - To identify the tests and checks | **Activity:** Cards matching game to discuss the test and checks and who to see.  See attached cards | Record Card – see attached appendix 2  NDSS Fact sheets:   - Annual Cycle of Care - Diabetes related complications - Sexual health and diabetes |
| **Simple steps to healthier feet** | - To understand the importance of a regular home foot inspection. - To demonstrate a foot inspection. - To recall the action to take if there is a change identified. | **Activity:** take off shoes and socks and inspect feet  **Activity:** rub oil/cream into one hand and compare to other  **Aides:** files, clippers, creams, mirrors  **Each person:** sample of Plunket’s products | NDSS Fact sheets:   - Looking after your feet |
| **Simples steps to healthier meals** | - To recall the role of healthier eating in self-managing their diabetes - To identify the simple steps to eating healthier - To demonstrate food selection for a healthy meal - To recall to find more information about food and diabetes | **Activity:** assemble a healthy plate meal using food models  **Demonstrate:** Sugar per serve – soft drinks, juice and compare to common food items (food model and packet foods)  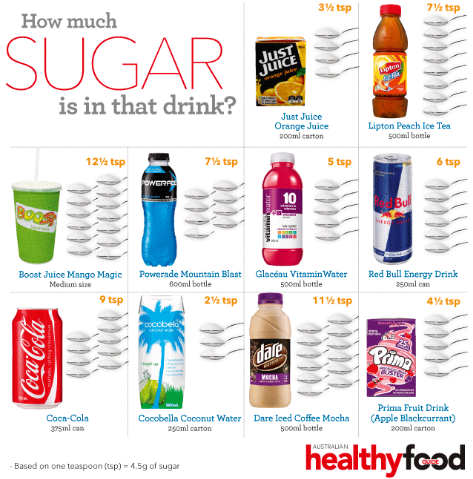 | NDSS Fact sheets:   - Healthy food choice - Healthy Meal Tips information sheet – see attached |
| **Simple steps to moving more** | - To recall the role of physical activity in self-managing their diabetes - To identify the simple steps to moving more - To identify one thing to start being more physically active - To recall where to find more information about physical activity | **Activity:** using the current exercise guidelines (Department of Health) – using attached activity outline (1) | NDSS Fact sheets:   - Physical Activity   Possible programs (NDSS free to attend if registered:   - Ready Set Go let’s Move - Beat it Gym |
| **Medications to insulin** | - To recall the role of medications in managing diabetes - To identify how their medication works, and possible side-effects - To recall where to find more information about medications | **Activity:** whiteboard: everyone identifies the medications they are taking. Compare the way each medication works, when to take and possible side-effects. | NDSS Fact sheets:   - Medications for type 2 - Insulin - Steroid Medication |
| **Sick days** | - To recall their role in managing sick days, hospital admissions | **Activity:** each person to develop a sick day plan using template provided following group discussion on what is needed in the plan.  **Equipment:** Whiteboard, pens  **Key discussion points:**   - Effect on BGL - What equipment is needed - Action to take | NDSS Fact sheets:   - Sick days and type 2 diabetes |
