# Supplementary material for: Shared Medical Appointments and Mindfulness for Type 2 Diabetes—A Mixed-Methods Feasibility Study
Source: Front Endocrinol (Lausanne). 2020 Oct 6;11:570777. doi: 10.3389/fendo.2020.570777 (PMC7573307; doi:10.3389/fendo.2020.570777)
Supplement: Supplementary file 2 [file Table_2.doc]

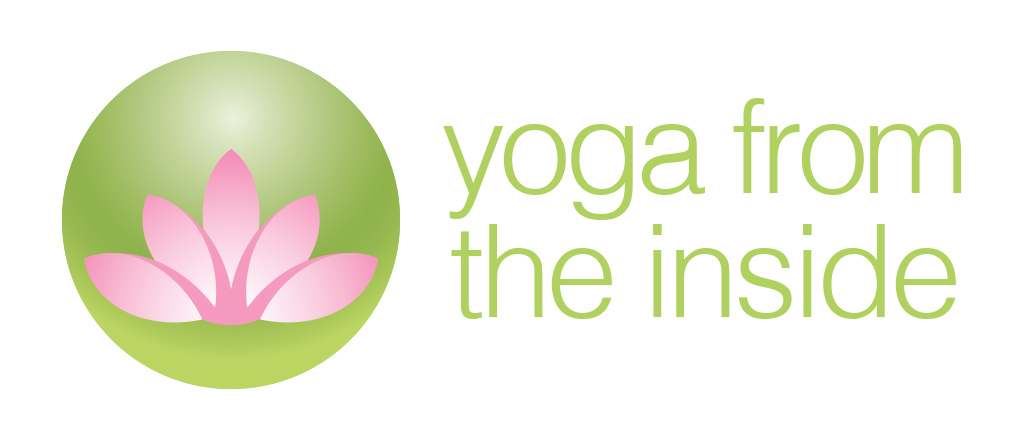
 **Catherine Sherlock**

**Outline for mindfulness component of SMA for diabetes clinical trial - NICM**

Each session will be 20 minutes comprised of five minutes to introduce a key mindfulness concept and a 15 minute practice. Participants will be seated comfortably in chairs, and inline with a trauma-sensitive protocol will be invited to keep their eyes open or closed depending on what they felt more comfortable with.

Participants will be encouraged to record (on their own device) the practice for their own use, between five and seven days per week. To be practiced at a time that works within their lifestyle. As educator I will also record the practice so that it is available to provide to anyone who may miss a session and for the purpose of documenting the trial.

The following table outlines the content of the theme of each session and the accompanying mindfulness practice.

| **Date** | **Concept or theme for discussion (5 mins)** | **Practice outline (15 mins)** |
| --- | --- | --- |
| 6/6/19 | Defining mindfulness:  *Mindfulness means paying attention in a particular way; on purpose, in the present moment, and non-judgementally*  Emphasis the two wings of mindfulness – awareness and compassion | Foundational body scan |
| 20/6/19 | Simply can’t do it wrong:  Just about noticing. Noticing = awareness  How is it possible to be aware in an incorrect way?  Introduce the concept of the witness You remain in control of your experience  You are the expert on you – you are your own teacher | Simple body scan and breath sensing |
| 4/7/19 | Finding an appropriate anchor:  Body, breath, sound, sensation, inner resource | Tone nervous system (simple body/breath sensing) and experiment with using chosen anchor |
| 18/7/19 | You are not your thoughts:  Observing the mind thinking itself  Not trying to get rid of thinking  Thinking not bad, but can be overused | Tone nervous system and then use anchor while observing the thinking mind |
| 1/8/19 | Emotions as sensations in the body | Tone nervous system then use anchor while exploring emotions |
| 15/8/19 | Welcoming/radical acceptance:  Everything as it is. Letting go of fixing and changing | Tone nervous system and offer resting in awareness practice, with anchor. |

In addition, the themes that were covered in each education component of the SMAs were woven into the mindfulness sessions as follows:

**Understanding and monitoring blood glucose levels** – Use mindfulness to develop awareness of the felt sense of changing glucose levels in the body – taking time to notice where and how it feels in the body when BGL are high/low.  This could be covered in discussion in relation to the informal practice of mindfulness at various times throughout the day in addition to a set time each day to sit/lie and practice.

**Feet –**noticing the effect on the body/mind when self-massaging feet. Suggest that feeling into the feet may be grounding, but noticing for themselves what happens when they feel into the sensation of their feet – what their experience is – noticing for anything else that may arise.

**Healthier meals –**notice any emotions, feelings, thoughts, shoulds or shouldn’ts that arise around suggestions about eating healthier meals.

**Moving more –**as above in relation to suggestions to move more

**Medication –**noticing any emotions, thoughts, feelings, stories that surface around medication

**Sick Days –**as above in relation to managing sick days.
